# Study of the Serum Metabolomic Profile in Nonalcoholic Fatty Liver Disease: Research and Clinical Perspectives

**DOI:** 10.3390/metabo8010017

**Published:** 2018-02-24

**Authors:** Stefano Gitto, Filippo Schepis, Pietro Andreone, Erica Villa

**Affiliations:** 1 Department of Medical and Surgical Sciences, University of Bologna and Azienda Ospedaliero-Universitaria di Bologna, Policlinico Sant’Orsola-Malpighi, 40138 Bologna, Italy; stefano.gitto2@unibo.it (S.G.); pietro.andreone@unibo.it (P.A.); 2 Research Centre for the Study of Hepatitis, University of Bologna, 40138 Bologna, Italy; 3 Department of Gastroenterology, Azienda Ospedaliero-Universitaria and University of Modena and Reggio Emilia, 41124 Modena, Italy; fschepis@unimore.it

**Keywords:** liver metabolomics, translational medicine, hepatic metabolism

## Abstract

In recent years, metabolomics has attracted great scientific attention. The metabolomics methodology might permit a view into transitional phases between healthy liver and nonalcoholic steatohepatitis. Metabolomics can help to analyze the metabolic alterations that play a main role in the progression of nonalcoholic steatohepatitis. Lipid, glucose, amino acid, and bile acid metabolism should be widely studied to understand the complex pathogenesis of nonalcoholic steatohepatitis. The discovery of new biomarkers would be important for diagnosis and staging of liver disease as well as for the assessment of efficacy of new drugs. Here, we review the metabolomics data regarding nonalcoholic fatty liver disease and nonalcoholic steatohepatitis. We analyzed the main studies regarding the application of metabolomics methodology in the complex context of nonalcoholic steatohepatitis, trying to create a bridge from the basic to the clinical aspects.

## 1. Introduction

Nonalcoholic fatty liver disease (NAFLD) represents the most common cause of chronic liver disease in the Western countries [1]. The clinical-histologic phenotype of NAFLD ranges from nonalcoholic fatty liver (NAFL) to nonalcoholic steatohepatitis (NASH). NASH is characterized not only by fat accumulation, but also by inflammation, ballooning degeneration, necro-apoptosis, and fibrosis. Notably, in about one third of patients, NASH can progress up to cirrhosis, hepatocellular carcinoma, and end-stage liver disease [2]. NASH represents a major clinical issue affecting about 1% of the European and North American population [3] and in recent years it has become one of the main causes for liver transplantation [4].

The alteration of glucose and lipid metabolism has a central role in the pathogenesis of NASH. In fact, patients with NASH often show insulin resistance (IR), central obesity, reduced glucose tolerance, type 2 diabetes mellitus (T2DM), arterial hypertension, and hypertriglyceridemia [5,6].

Liver biopsy is the gold standard for the diagnosis of NASH [7]. However, the invasiveness and the sampling variability are major unresolved issues [7]. Indeed, it is not surprising that many researchers have proposed some non-invasive markers of both steatosis and fibrosis [8].

The lack of a widely approved therapeutic approach represents another major concern. Today, the standard of care for the treatment of NASH is lifestyle improvement [2]. However, the effect of lifestyle intervention on liver histology needs further evaluation, as there is a lack of evidence about the optimal dietary nutrient composition and exercise requirement. On the other hand, vitamin E and pioglitazone are the only approved pharmacological options for patients with NASH. Nevertheless, there are noteworthy limits concerning efficacy and long-term safety [2].

In this scenario, the discovery of new biomarkers of NAFLD/NASH useful for diagnosis, staging, and assessment of efficacy of new drugs is an important scientific goal.

Metabolomics might be an interesting tool for this aim since it can be applied for the early diagnosis of liver disease, for better understanding the pathogenesis, and for the development of new drugs [9]. Metabolomics, with other “omics” technologies, might support the accurate understanding of biochemical events inside the cells. Metabolomics represents a new and powerful technology for biomarker detection within a dynamic field. Together with genomics, transcriptomics, and proteomics, metabolomics can lead to a wide knowledge of biological systems [9].

Although the term “metabolomics” and the related techniques have been only recently adopted, some simple applications of the metabolomics concept date back to many centuries ago. An example is the “urine wheel”, published in 1506 by Ullrich Pinder in the book entitled *Epiphanie Medicorum* [10].

The liver represents the main source of many endogenous metabolites, precursors, and detoxification enzymes [11]. The liver metabolome includes a complex and dynamic flux of small metabolites (by definition <1.5 kDa). Mass spectrometry (MS)-based techniques such as gas chromatography/MS, liquid chromatography (LC)/MS, nuclear magnetic resonance (NMR) spectroscopy, and Fourier transform infrared spectroscopy (FTIR), represent the applied techniques for an appropriate metabolomics analysis. Notably, the different technologies enable one to obtain a wide view of metabolite profiles [12].

In the clinical practice, the detected and quantified biomarkers can be used as unbiased indicators of illness onset and progression and as support for disease classification. Biomarkers might become prognostic indicators that could be used for risk stratification of the general population. Moreover, the metabolomics approach might be useful for the early diagnosis of liver disease and for assessing the efficacy of new therapeutic interventions.

Herein, we describe the main animal and human studies concerning the application of a metabolomics approach in the NAFLD context. We conducted a complete PubMed Literature search using the key terms “non-alcoholic fatty liver disease”, “non-alcoholic steatohepatitis”, as well as the name of each known biomarker. Articles indexed between 2000 and 2017 were analyzed. We examined some crucial metabolic pathways involved in liver disease, including altered key metabolic pathways such as cellular energy metabolism, lipid and glucose metabolism, amino acid pathways, metabolism of bile acids (BAs), and oxidative stress.

## 2. Metabolic Alterations of NAFLD

### 2.1. Modification of Lipid Metabolism

It is well known that NAFLD is characterized by significant increase of lipid species in both the blood and liver tissue. Disruption of lipid balance consists of changes in cholesterol esters, triacylglycerols (TG), diacylglycerols, and sphingomyelins [13,14,15,16]. Puri et al. [17] compared plasma lipids and eicosanoid metabolites quantified by MS in three subgroups: simple NAFL, NASH, and healthy controls (HC). Both patients with NAFL and subjects with NASH showed a noteworthy rise of total monounsaturated fatty acids due to palmitoleic and oleic acids and an increase of gamma-linolenic and dihomo gamma-linolenic acids. Notably, palmitoleic acid, oleic acid, and the palmitoleic acid to palmitic acid ratio were considerably augmented in patients with NAFL.

The increased turnover of phospocholine (PC) and phosphatidylethanolamine species in the liver represents another important topic emerging from NAFLD/NASH animal models. Interestingly, it determines the release of free fatty acids through the action of phospholipases A1 and A2 that are not catabolized by β-oxidation, and therefore get stored in the liver as TG [13,14,15].

Remarkably, fatty liver accumulation is not just a fat deposition in the liver but rather a “reorganization” of lipid storage [13]. Van Ginneken et al. [13], using a 24-h starvation mice protocol, demonstrating that adipose tissue was the main source of liver TG. They demonstrated that animals with fatty liver showed increased hepatic concentrations of lysophosphatidylcholine (LPC), lysophosphatidylethanolamine (LPE), and PC species. [18]. On the other hand, Pereira et al. [19] reported that obese female Sprague–Dawley rats developed an increase in diacylglycerol and a drop in phosphatidylethanolamine in comparison with lean animals. Moreover, the expression of both phosphoethanolamine cytidylyltransferase and peroxisomal proliferator activated receptor-α mRNA was lessened in the hepatic tissue of obese rats compared to the others.

Kalhan et al. [16] developed a metabolomics study enrolling 11 non-diabetic subjects with histologically proven steatosis, 24 with NASH, and 25 healthy controls (HC). Many free fatty acids such as eicosopentaenoate, docosohexaenoate, 10-undecenoate, and arachidonate were significantly lower in patients with NASH in comparison with HC. Notably, only caprate and 10-undecenoate were significantly lower in subjects with NAFL in comparison with HC. Finally, linolenate and undecanoate were considerably increased in subjects with NAFL in respect to NASH.

Interestingly, Tu et al. [20] tried to clarify the pathogenesis of non-obese NAFLD that definitely represents clinical unresolved issues. They demonstrated that systemic mechanisms leading to steatosis and liver damage in the non-obese mouse model were due to a mixture of effects of elevated free cholesterol, cholesterol esters, and cholic acid, and related modifications of sphingomyelin and phosphatidylcholine metabolism. Remarkably, the authors described one of the first metabolite reference profiles useful for understanding the properties of dietary and hepatic cholesterol in non-obese human NAFLD subjects.

Papandreou et al. [21] analyzed the rearrangement of lipid biosynthesis occurring in patients with non-alcoholic liver steatosis with a follow-up period of 3.8 years. They subdivided 45 patients into the following subgroups: (1) HC; (2) liver steatosis; (3) liver steatosis reversion during the follow-up. At baseline, patients with liver steatosis displayed lesser storage and transport of TG and cholesteryl esters, several monoetherglycerophosphocholines, acylglycerophosphocholines, ceramides, and ceramide to sphingomyelin ratio in comparison with HC. P-ether acylglycerophosphocholines, ceramides, and sphingolipids were significantly dissimilar between group 3 and group 1. Interestingly, the alterations of metabolic profile were found not only in patients with liver steatosis but also in patients with liver steatosis reversion, probably because the negative metabolic condition required a long time period for complete regression.

### 2.2. Glucose Metabolism

In animal models of NASH, glucose concentrations can be decreased in both the serum [22] and liver tissue [23]. Toye et al. [24] analyzed glucose tolerance, in vivo insulin secretion, the plasma lipid profile, and adiposity of mice with high fat diet-induced NAFLD. According to the authors, the increased plasma lactate concentrations in fat-fed mice might indicate muscle lactate production due to hyperglycemia. In general, NAFLD seems to favor the cytosolic glycolysis with a rise of serum/plasma lactate, pyruvate, and alanine [24]. Notably, the onset of IR represents one of the main negative turning points in the pathogenesis of NAFLD and NASH [2]. In the mouse model of liver steatosis, the rise of insulin levels and the associated activation of pyruvate kinase M2 can favor glycolysis and the consequent increased production of lactate and alanine from glucose via pyruvate [25]. Kalhan et al. [16] showed that glucose and pyruvate were markedly higher in subjects with NASH. Mannose, lactate, and erythronate levels were more pronounced in both steatosis and NASH than HC.

### 2.3. Amino Acids

Patients with liver disease usually show wide modification of essential amino acids and common branched-chain amino acids (such as valine, leucine, and isoleucine). Leucine, isoleucine, and valine might mediate activation of several important hepatic metabolic signaling pathways ranging from insulin signaling to glucose regulation [9]. It is therefore not surprising that they have a significant role in the management of NAFLD and NASH.

García-Canaveras et al. [18] reported that in patients with NAFLD in contrast to the others, an impaired recycling of amino acids could be found. This imbalance of amino acid homeostasis might determine an alteration of glutamate, glutamine, and glutamyl-peptide.

Kalhan et al. [16] described a rise of glycochenodeoxycholate, glycocholate, and taurocholate in the subgroup of patients with NAFL. In patients with NASH, free carnitine, butyrylcarnitine, and methylbutyrylcarnitine increased while long-chain fatty acids decreased. The cysteine-glutathione level was lower in NASH and NAFL, while glutamyl dipeptides were higher in comparison with HC. Notably, it was demonstrated that in patients with NAFLD, cysteine-glutathione disulfide and oxidized/reduced glutathione declined in both liver tissue [18] and blood [16], representing a clear sign of active oxidative stress.

Goffredo et al. [26] analyzed the disorders of amino acid metabolism in obese young people with NAFLD. The metabolomics signature of 78 obese adolescents with or without NAFLD was analyzed. Subjects with NAFLD showed higher plasma levels of valine, isoleucine, tryptophan, and lysine. Interestingly, it was demonstrated that a higher baseline valine level could represent a strong predictor of major hepatic fat accumulation.

Kaikkonen et al. [27] proposed another study about NAFLD in young adults. Interestingly, they focused on the metabolic perturbations preceding the onset of liver disease. Sera from the population-based Young Finns Study were analyzed (year 2001, *n* = 1575; year 2007, *n* = 1509; year 2011, *n* = 2002). The presence of NAFLD was assessed in 2011. Extremely large very-low-density lipoprotein TG, very-low-density lipoprotein measures, and branched-chain amino acids were strongly associated with the onset of liver disease. Notably, high-density lipoprotein measures and several fatty acids including omega-6 were inversely related to NAFLD development.

### 2.4. Bile Acids

BAs are important signaling molecules for both lipid and glucose homeostasis. Chenodeoxycholic and deoxycholic acids are endogenous ligands of farnesoid X receptor (FXR) that is a key metabolic receptor [28]. In the liver, FXR modulates the conversion of cholesterol to BAs by regulation of the expression of cytochrome p450 7A1. FXR also induces the expression of the canalicular bile salt export pump and multidrug resistance-associated protein 2. In the ileum, FXR receptor decreases the uptake of BAs through a down-regulation of the sodium-dependent BA transporter. Furthermore, FXR improves expression of ileal BA-binding protein, which acts as a shuttling device for the transport of BAs to the basolateral membrane of erythrocytes. Remarkably, FXR inhibits both the hydroxymethylglutaryl-CoA reductase and the lanosterol 14 α-demethylase that play central roles in cholesterol synthesis [28]. A four-fold increase in the plasma concentration of glycocholate and taurocholate, and a two-fold increase in glycochenodeoxycholate were found in patients with NASH vs. HC [16]. These BAs were also higher in patients with steatosis compared with HC (but only taurocholate reached statistical significance). García-Canaveras, et al. [18], using a LC-MS-based metabolomics analysis, evaluated 23 steatotic and 23 non-steatotic human livers. It was confirmed that patients with steatosis showed higher levels of BAs and phospholipid degradation products compared to the others.

## 3. Metabolomics Profile Modifications: NAFL vs. NASH

### 3.1. Animal Studies

As indicated in the previous paragraphs, NASH represents the most dangerous expression of the NAFLD spectrum [2]. Notably, only a few metabolomics studies have investigated the complex mechanisms leading to the progression from simple NAFL toward NASH and NASH-cirrhosis.

Li et al. [22] used a methionine and choline deficient (MCD) diet to describe different stages of NAFLD in male C57BL/6 mice. With a proton nuclear magnetic resonance approach, the showed that betaine, PC, choline, and trimethylamine *N*-oxide were up-regulated in both the liver and plasma of fatty liver rodents. Serum glucose, glutamate/glutamine, lactate, and taurine were identified as biomarkers of NAFLD. Furthermore, a model was built to predict the NAFLD progression rate based on the different levels of these four biomarkers.

Tanaka et al. [29] analyzed NASH pathogenesis, combining high-end analytics and targeted gene expression. They induced a NASH-like disease in mice fed a MCD diet and compared them with normal diet animals. They demonstrated a significant decrease of LPC with a related rise in tauro-β-muricholate, taurocholate, and 12-hydroxyeicosatetraenoic acid. Moreover, it was suggested that the decrease of LPC and the increase of serum BAs could represent a sign of inflammation. In fact, phospholipid and BA metabolism was altered in NASH but not in simple steatosis.

Zhao et al. [30] further investigated the modifications of LPC and BA homeostasis in the NASH model. In particular, they quantified the hepatic mRNA levels related to genes involved in the metabolism and transport of LPC, Bas, and 12-hydroxyeicosatetraenoic acid. In mice with NASH, LPC acyltransferases (LPCAT) (that convert LPC to PC) were up-regulated by two- to four-fold. The above reported findings might clarify how the inflammatory phenotype of NASH in the mouse model results in the variations of serum metabolites. Notably, similar alterations have been described patients with NASH [16], suggesting that similar mechanisms can operate in humans.

Recently, Alonso et al. [31] analyzed liver tissue and serum from methionine adenosyltransferase 1a knockout (MAT1A-KO) and C57Bl/6 (controls) mice. Animals showed chronically low levels of hepatic S-adenosylmethionine (SAMe), which plays a key role in the maintenance of cellular homeostasis and cell cycle control, and in NASH-like liver disease. It was demonstrated that deletion of MAT1A, an integrator of the cellular metabolic status, might not only affect hepatic metabolism downstream of SAMe, but also leads to an imbalance in the circulation of one-carbon units from specific amino acids to folates, thus altering the biosynthesis of lipids, proteins, and amino acids, and mitochondrial polarization and function. Notably, it was suggested that the described changes might favor NASH onset. Indeed, altered SAMe level might be a significant determinant that might cause the switch from NAFL to NASH. Remarkably, the authors’ conclusions are coherent with the finding that NASH patients with advanced liver disease tend to develop a decreased MAT1A expression [32].

In contrast with Alonso et al. [31], Del Bas et al. [33] demonstrated that animals with metabolic syndrome and NASH developed an unexpected accumulation of hepatic SAMe. Consequently, progression of NAFLD in the context of metabolic syndrome could happen even in a state of hepatic SAMe excess.

In this complex context, Liu et al. [34] performed an extensive study of different NAFLD phenotypes, using a high-fat diet, a MCD diet, and streptozocin in different rat models to compare NAFL, NASH, and NAFL + T2DM. Transcriptomics and metabolomics tests were conducted in both liver tissue and serum samples. Ninety-six genes, 17 liver metabolites, and 4 serum metabolites reliably changed. Notably, stearoyl-carnitine was the only metabolite modified in both liver and serum of the three NAFLD phenotypes. The analyzed genes and metabolites showed a noteworthy role in lipid, glucose, and fatty acid metabolism and in the immune and inflammatory response.

### 3.2. Human Studies

Kalhan et al. [16] conducted a small study analyzing data from 11 non-diabetic subjects with histologically proven steatosis, 24 with NASH, and 25 HC. Notably, only some metabolites were significantly different in the plasma of subjects with NAFL and NASH. Glutamate, creatine, pyruvate, unknown X-01911_200, were lower in patients with NAFL compared to NASH, while undecenoate and linolenate were considerably higher.

Puri et al. [17] presented a study comparing plasma lipids and eicosanoid metabolites quantified by MS in patients with NAFL or NASH versus HC. Notably, NASH patients showed a reduction in the docosahexanoic acid to docosapentenoic acid ratio and a rise in the level of 11-hydroxyeicosatetraenoic acid (arachidonic acid’s derivate).

Barr et al. [35] developed a translational study with both humans (NAFLD and HC) and an animal model (Gnmt-KO mice) using a global metabolite profiling methodology. In NAFLD samples, the levels of organic acids, PC, LPC, arachidonic acid, glutamic acid, Bas, and sphingomyelin lipids were significantly altered. The amount of deoxycholic acid was significantly higher in NAFLD versus normal liver. A further analysis was dedicated to find the differences between human NAFL and NASH. Significant changes in serum concentrations were found in antioxidative ether glycerophospholipids, sn-2 arachidonly diacylglycerophosphocholine, and free arachidonic acid (the latter two with a relevant role in eicosanoid signaling pathways). Interestingly, the same first author [36] studied a large sample size (467 biopsied individuals with normal liver histology, NAFL, or NASH), observing comparable phenotypes in humans and mice with NASH, such as reduced levels of poly-unsaturated fatty acids, sphingomyelin, and a number of LPC species. In patients with NASH, high acyl carnitines (sign of mitochondrial β-oxidation dysfunction), altered ether phospholipids, and boosted proinflammatory eicosanoids can be detected.

Han et al. [37] proposed another translational human-animal study. In this study, the main metabolic pathways associated with progression of NASH were analyzed. They reported that metabolome changes in both humans and the rat model of NASH mainly concerned amino acid and BA metabolism. Notably, the study underlined some relevant differences between the metabolomics data from animals and humans, mainly regarding the biochemical pathogenesis of NASH. Taurine, an organic acid that conjugates BAs, was considerably boosted in human NASH but not in the animal model. Moreover, betaine and creatine exhibited reverse modification between animals and humans. Indeed, selected essential amino acids could be potential biomarkers for liver disease progression and the mixture of metabolomics variations of BAs, amino acids, and fatty acids could help to better understand the pathogenesis of NASH.

Recently, Dong et al. [38] identified some biomarkers that may be different according the NAFLD stage. They analyzed urine and blood of the following subgroups of patients: non-diabetic patients with NAFLD and normal liver function (*n* = 33), NASH with impairment of liver function (*n* = 45), and HC (*n* = 30). Compared to the HC, patients with NASH displayed higher urinary levels of lysine, valine, citrulline, arginine, threonine, tyrosine, leucine, hippuric acid, and 3-indoleacetic acid but also decreased levels of derivatives of indole acetic acid, such as 5-hydroxy indole acetic acid and indole-3-formic acid. Moreover, their cortisol level considerably dropped. It was demonstrated that 2-methylguanosine, gluconic acid, indoxylsulfuric acid, cAMP, indolelactic acid, and acetyl-DL-leucine were able to discriminate patients with NASH and HC. Patients with NASH showed higher levels of methyl xanthine, tryptophan, 3-indole acetic acid, and gluconic acid, and lower proline. As demonstrated by the ROC analysis, 3-indoleacetic acid, l-carnitine, pyroglutamic acid, and indolelactic acid might differentiate NASH from NAFLD.

Lake et al. [39] analyzed the complex progression of steatosis toward NASH by metabolomic and transcriptomic analysis. They reported rises in the levels of leucine (+127%), isoleucine (+139%), and valine (+147%) from NAFL to NASH. Also, carnitine metabolites were significantly elevated in NASH compared to NAFL.

Sokooian et al. [40], in a two-stage case-control study, included patients with biopsy-proven NAFLD and investigated the circulating levels of betaine and its association with the histological spectrum. Betaine level correlated with the disease severity and with liver inflammation, ballooning degeneration, and fibrosis. Betaine concentration was considerably decreased in NASH patients in comparison with NAFL. The role of betaine in the modulation of the methylome is relevant as the process of methylogenesis is exclusively active in the liver that represents a large betaine reservoir.

O’Sullivan et al. [41] used an integrated, non-targeted metabolomics, genetic, and human phenotyping study to analyze the role of dimethylguanidino valeric acid (DMGV) as a biomarker of NAFLD in individuals undergoing gastric bypass surgery. Plasma DMGV levels showed a relevant correlation with biopsy-proven NASH. In detail, DMGV levels fell in parallel with improvements of cardio-metabolic parameters. Remarkably, the baseline DMGV level was an independent predictor of long-term T2DM. The main patterns of the human-based available studies are summarized in Table 1.

## 4. Integrated Clinical Points of View

To gain real insight in the pathogenesis of human liver damage and in the progression of NAFLD, experimental data need to be bridged to the clinical condition. At least three studies have been performed toward this goal with noteworthy results.

Calvo et al. [42] verified in an observational, prospective, single-site, 1-year cross-sectional study showing the usefulness of magnetic resonance imaging (MRI) and spectroscopy (MRS) for assessment of NAFLD in patients undergoing bariatric surgery. They utilized the H NMR-based metabolomics approach. MRI data showed a pronounced relationship with the histological assessment (such as liver TG). The increased levels of TG and monounsaturated fatty acids in severe steatosis were associated with a substantial reduction in diglycerides, polyunsaturated fatty acids, glucose-6-phosphate, and the ATP/AMP ratio.

Zhou et al. [43] proposed a large study (318 subjects) comparing the MS-based profiling of plasma, the routinely available clinical parameters, and the patatin-like phospholipase domain-containing protein 3 (PNPLA3) genotype as potential tools for diagnosis of NASH. They developed the NASH ClinLipMet Score that includes glutamate, isoleucine, glycine, LPC, phosphoethanolamine, AST, and fasting insulin, along with PNPLA3 genotype. The “NASH ClinLipMet” score was able to identify patients with NASH with a relevant ROC value [0.866 (95% confidence interval, 0.820–0.913)], which was significantly higher than “NASH Clin Score” [0.778 (95% confidence interval, 0.709–0.846)].

More recently, Del Chierico et al. [44] analyzed the gut microbiota of pediatric NAFLD patients with both metagenomics and metabolomics methodologies. They enrolled 61 patients with NAFL, NASH, or obesity, and 54 HC. In comparison with HC, a decrease of *Oscillospira* in NAFL and NASH groups and an increase in species such as *Ruminococcus*, *Blautia*, and *Dorea* was detected. The study of *Oscillospira*, *Rickenellaceae*, *Parabacteroides*, *Bacteroides fragilis*, *Sutterella, Lachnospiraceae*, 4-methyl-2-pentanone, 1-butanol, and 2-butanone might help to categorize patients at risk of NAFLD/NASH. In NASH subjects, lower levels of *Oscillospira* were associated with higher levels of *Dorea* and *Ruminococcus*. It was suggested that the interrelated enterotype-metabotype framework might help to discover a specific signature of disease progression. Notably, the relevant amount of species such as *Lachnospiraceae*, *Ruminococcus*, and *Dorea* found in pediatric patients with NASH might indicate that some modifications of microbiota can constitute a significant risk factor for liver disease progression.

## 5. Conclusions and Future Perspectives

The pathogenesis of NASH is complex since many events occur in parallel. IR, adipocyte proliferation, and changes in the intestinal flora seem to have a relevant role. Adipokines such as IL-6 and TNF-α produced by adipocytes can influence the hepatocyte fat content, favoring liver tissue inflammation. Free fatty acids and free cholesterol induce endoplasmic reticulum stress and oxidative stress resulting in hepatic inflammation and fibrogenesis [2]. This complexity at least partially explains the lack of a consolidated therapeutic approach and the difficulties in diagnostic assessment.

Metabolomics might be one of the most stimulating ways to analyze the metabolic alterations associated with NASH [9]. The quickly expanding research field of metabolomics involves the study of the molecular species smaller than <1.5 kDa in any fluid, cell, or tissue [45,46,47].

The liver shows some peculiar patterns that make it the ideal context for metabolomic study. In fact, no other organ or tissue processes such a plethora of lipids and water-soluble substances [11].

Many authors have proposed non-invasive markers of liver disease that might discriminate the early stages of damage, thus helping clinicians in the diagnostic process [8]. Dong et al. [38] worked in this direction with an interesting study. They developed a panel of urinary biomarkers that might help to discriminate between NAFL and NASH. The comparison between NAFL and NASH showed that the most relevant differences were about nucleic acids and amino acids that represent an adaptive physiological response to liver stress. The level of cholinesterase was significantly lower in patients with NASH in comparison with NAFL. Low cholinesterase levels can have a negative impact on the synthesis and secretion of very low-density lipoprotein. Remarkably, it was also demonstrated that indoleacetic acid was higher in patients with NASH in comparison with NAFL. Notably, the major differences between NAFL and NASH concern energy impairment. Indeed, future research studies might go in this direction. Unfortunately, many critical points can be registered. First of all, gender, ethnicity, age, nutritional status, and presence of comorbidities might lead to significant differences in the single molecule threshold of normality [48]. The identification of patients at risk for NASH development and progression remains an unresolved challenge.

A greater understanding of the evolution of NAFLD/NASH should evaluate the role of lipid, glucose, amino acids, and BAs metabolism. The study of biomarkers associated to the main metabolic process involved in NASH pathogenesis might lead to the development of new therapeutic options.

In general, the most important issue for an effective prevention program depends on the early detection, treatment, and monitoring of high-risk subjects. This aim can be reached through the quantification of the disease-specific biomarkers summarized in this review.

In this complex context, a translational approach might be more effective. An important translational study with animal models and humans was developed by parallel analyses among Gnmt-KO mice, NAFLD patients, and HC [35]. In NAFLD samples, the levels of organic acids, PC, LPC, arachidonic acid, glutamic acid, Bas, and sphingomyelin lipids were significantly different in comparison with HC. Many differences in antioxidative ether glycerophospholipids, sn-2 arachidonly diacylglycerophosphocholine, and free arachidonic acid emerged between NAFL and NASH.

The possible application of the cited studies in clinical practice represents a main issue. Indeed, the study by Zhou et al. [43] and the development of NASH ClinLipMet score is an important turning point and should be a model for further studies.

Another main future perspective concerns the possible prevention of hepatocellular carcinoma (HCC) onset. Although this is not the principal aim of the present review, we would like to cite the study by Teilhet et al. [49]. They performed a comparative metabolomics study between patients with NASH-related cirrhosis and non-cirrhotic NAFLD who underwent hepatectomy for HCC. The study included 28 pairs of HCC samples versus Non-Tumoral Tissue (NTT). In HCC tissue, compared to NTT, high levels of lactate and phosphocholine, and low glucose were detected. High levels of β-hydroxybutyrate, tyrosine, phenylalanine, and histidine were revealed in patients with cirrhosis compared to non-cirrhotic ones. On the contrary, major levels of glutamine/glutamate were described in non-cirrhotic patients in comparison with the others.

The available studies showed that there are clear differences in the metabolomic patterns of healthy subjects and patients with NAFLD. However, to differentiate patients with NAFL and those with NASH remains a true challenge. Actually, only a few metabolites were significantly different between NAFL and NASH. In the future, it would be necessary to develop large cohort studies with the aim of evaluating the metabolomics and histological differences between NAFL and NASH in parallel.

In the future, biomarkers might become important in every-day clinical practice, especially for the early diagnosis of high-risk patients. Moreover, the metabolomics approach could favor the development of personalized behavioral, diet, and drug-based therapy.

## Figures and Tables

**Table 1:** Main patterns of human-based Metabolomics studies about NAFLD.

First Author	Sample Size #	Method	Main Findings	Reference
Kalhan	**60**	**LC; GC; MS**	(1) Glutamate, creatine, pyruvate, unknown X-01911_200, were lower NAFL than NASH; (2) undecenoate and linolenate higher	[16]
Puri	**125**	**MS**	(1) Patients with NASH exhibited a decrease of docosahexanoic acid to docosapentenoic acid ratio and a rise of 11-hydroxyeicosatetraenoic acid than NAFL and HC	[17]
Barr	**42**	**LC; MS**	(1) Deoxycholic acid was higher in NAFLD versus normal liver; (2) antioxidative ether glycerophospholipids, sn-2 arachidonly diacylglycerophosphocholine, and free arachidonic acid were different between NAFL and NASH	[35]
Barr	**467**	**LC; MS**	(1) High serum NEFA in NAFLD patients; (2) Low NEFA and elevated acyl carnitines in NASH	[36]
Han	**58**	**LC; MS**	(1) The short-chain carnitines were higher in NASH; (2) long-chain fatty acid and phospholipids lower in NASH respect to the others; (3) significant dissimilarities between human NAFLD progression and rodent models	[37]
Dong	**108**	**LC; MS**	(1) Carnitine in urine was lower in NAFLD patients than in HC; (2) amino acids were higher in patients with NASH patients than in HC; (3) cholinesterase was lower in patients with NASH than NAFL; (4) level of indoleacetic acid was higher in the NASH group compared with NAFL	[38]
Lake	**45**	**LC; MS**	(1) NASH patients show higher levels of leucine, isoleucine, valine in respect to NAFL; (2) carnitine metabolites were higher in NASH than NAFL	[39]
Sokooian	**99**	**LC; MS**	(1) Patients with NASH showed a 1.26-fold decrease in betaine levels than NAFL	[40]
O’Sullivan	**470**	**LC; MS**	(1) DMGV levels increased in the presence of NASH; (2) DMGV levels fell in parallel with metabolic improvements	[41]

LC, liquid chromatography; MS, mass spectrometry; GC, gas chromatography; NAFL, Non-Alcoholic Fatty Liver; NASH, Non-alcoholic Steatohepatitis; HC, Healthy Controls; NEFA, Non-esterified fatty acids; NAFLD, Non-Alcoholic Fatty Liver Disease; DMGV, Dimethylguanidino valeric acid. # Number of human subjects involved (parallel animal models are not computed).
